# Effect of Wet-Dry Cycles on the Mechanical Performances and Microstructure of Pisha Sandstone

**DOI:** 10.3390/molecules28062533

**Published:** 2023-03-10

**Authors:** Yanbing Zhao, Caiqian Yang, Feng Qu, Zhiren Wu, Kejie Ding, Zhishui Liang

**Affiliations:** 1 Key Laboratory of Concrete and Prestressed Concrete Structures of Ministry of Education, Southeast University, Nanjing 210096, China; 2 Jiangsu ZYZ Intelligent Operation & Maintenance Institute, Nanjing 210003, China; 3 School of the Environment and Safety Engineering, Jiangsu University, Zhenjiang 212013, China

**Keywords:** Pisha sandstone, microstructure, mineral compositions, uniaxial compressive strength, wet–dry cycles

## Abstract

The effects of the wet–dry cycles on the chemical compositions, microstructure, and mechanical properties of Pisha sandstone were experimentally investigated in the current study. A series of uniaxial compression tests were conducted to validate the deterioration of the mechanical property of specimens after wet–dry cycles. In addition, the evolutions of the mineral compositions and microstructure characteristics were confirmed by X-ray diffraction (XRD) and scanning electron microscope (SEM). Experimental results indicated that with the increase of wet–dry cycles, the mechanical properties of Pisha sandstone gradually decrease. After five wet–dry cycles, the uniaxial compressive strength, elastic modulus, and fracture energy of specimens were reduced by 41.06%, 62.39%, and 31.92%, respectively. The failure mode of the specimen changes from inclined shear failure to peel failure. Compared to the initial specimens, the relative content of primary minerals after five wet–dry cycles declined by 5.94%, and the relative content of clay minerals after five wet–dry cycles increased by 54.33%. Additionally, the porosity of samples exhibits a positive correlation with wet–dry cycles. Compared to the initial specimens, the porosity of specimens after five wet–dry cycles increased by 176.32%. Finally, a prediction model of the correlation between uniaxial compressive strength and porosity is proposed and verified.

## 1. Introduction

Pisha sandstone is a typical loose layer composed of sandstone, arenaceous shale, and mudstone, which widely distributes around the border areas of Shanxi, Shaanxi, and Mongolia in the Yellow River Basin [1]. The Pisha sandstone is mainly white, grayish-white, and purplish-red in color and is generally layered. Pisha sandstone mainly consists of kaolinite, quartz, feldspar, muscovite, hematite, calcite, biotite, illite, and chlorite. Due to the intrinsically low diagenesis and inferior mechanical intensity, Pisha sandstone quickly turns into mud when encountering water [2]. Pisha sandstone is prone to disintegration and collapse under the action of external forces, such as freezing and thawing, gravity, wind, and water force [3,4,5]. Therefore, rainfall intensity and environmental humidity inevitably accelerate the erosion rate of the sandstone [6,7] and cause severe soil erosion problems. It has been reported that the modulus of soil erosion in the area of Pisha sandstone is approximately 30,000~40,000 t/(km^2^a), of which the annual average amount of sediment entering the Yellow River is nearly 200 million t, and the coarse sediment deposited can reach up to 100 million tons [8]. A total of 66.5% of the Pisha sand region has a large production of coarse sediment (d ≥ 0.05 mm), which provides 25% of the total sediment of the lower Yellow River [9,10]. Therefore, the Pisha sandstone area is widely regarded as the most severe soil erosion region on the Loess Plateau [9].

Previous studies revealed the impacts of rainwater infiltration on the slope stability [11,12] and microstructure [13] of Pisha sandstone. The erosion mechanisms of rainfall can be briefly summarized as follow: (1) water flow can result in surface, and fine trench erosion [14,15], and (2) the permeation of rainwater also compromises the structural performance [16] of Pisha sandstone. However, the interaction between sandstone and water not only involves the scouring and infiltration of rainfall. In a complex and diverse natural environment, tremendous factors may affect water–sandstone interaction, such as frequent rainfall and evaporation, the rise and fall of underground water, and the change of relative environmental humidity. That is, the sandstone inevitably undergoes the alternate action of dry and wet conditions, as well as repeated water absorption and dehydration phenomenon [17,18]. Nevertheless, such critical issues were squarely out of the scope of the abovementioned investigations. Limited correlated studies mainly concentrate on the properties of the reconstituted Pisha sandstone instead of original samples [19,20,21], which may be entirely unable to reveal the performances of Pisha sandstone under natural conditions.

The effects of the wet–dry cycles on the performances of unweathered Pisha sandstone were experimentally investigated in the current research study. A series of X-ray diffraction (XRD) and scanning electron microscope (SEM) experiments were conducted to reveal the changes in chemical compositions and physical properties of the samples during these stages. The failure modes, uniaxial compressive strength, elastic modulus, and fracture energy of Pisha sandstone at different wet–dry cycles were systematically compared and analyzed. Furthermore, the pore morphology parameters (average pore diameter and pore area) were carried out to quantify the deterioration trends of pore structure. Finally, a prediction model of the correlation between uniaxial compressive strength and porosity is proposed and verified.

## 2. Results and Discussion

### 2.1. Mechanical Performances

#### 2.1.1. Failure Modes

**Figure 1:**
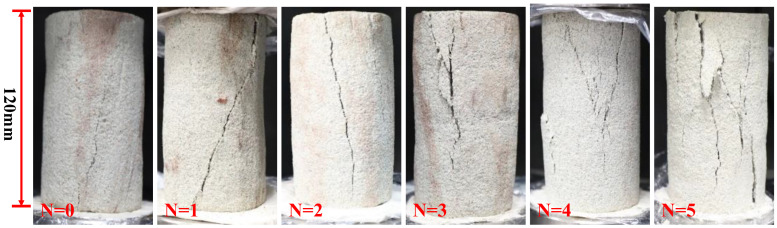
Failure modes of specimens at different wet–dry cycles.

The failure modes of specimens at each wet–dry cycle are plotted in Figure 1, in which the N represents the number of wet–dry cycles. The failure mode of the virgin samples is an inclined shear failure. The inclination angle between the primary crack and the axis of the Pisha sandstone was approximately 25–35°. In the early stage of the wet–dry cycle (≤2), the failure mode is still the inclined shear failure. However, the width of the primary crack increases and the number of cracks increase. In addition, the inclination angle is gradually reduced. At the later stage of the wet–dry cycle (>2), the primary crack no longer extends diagonally, the failure modes of specimens transition to peel failure, the cracks increase significantly, and the side surfaces of the samples begin to peel off. This phenomenon demonstrates that wet-dry cycling action has a significant effect on the failure modes of Pisha sandstone.

#### 2.1.2. Water Absorption

**Figure 2:**
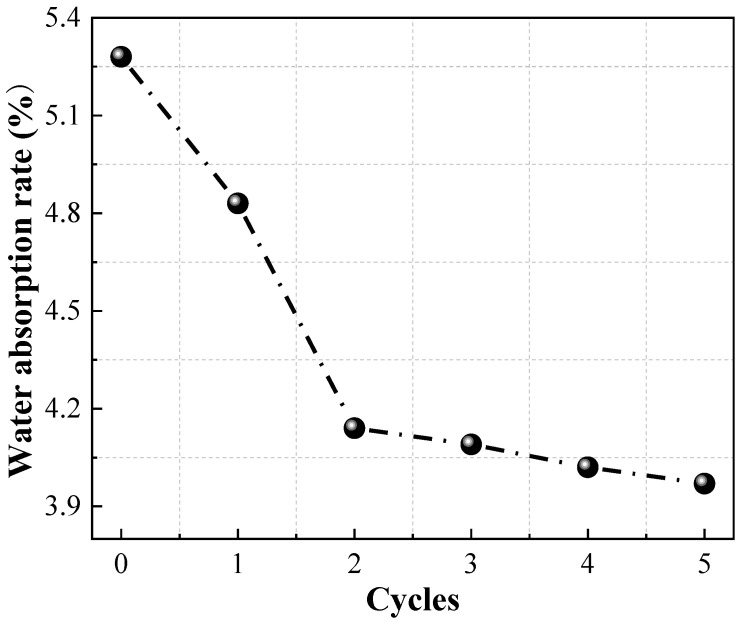
Water absorption rate of Pisha sandstone specimens at different wet–dry cycles.

The macro-mechanical properties of rocks with different water contents vary greatly, and the investigation of water absorption properties of Pisha sandstone under different wet–dry cycles is of great significance to studying the weathering and erosion mechanism of Pisha sandstone. Therefore, the water absorption performance of Pisha sandstone specimens after different dry–wet cycles was measured, as shown in Figure 2. The formula for calculating the water absorption rate is displayed in Equation (1), where W*_N_* is the water absorption rate after N dry–wet cycles; M*_N_*_1_ is the mass of the specimen when it is saturated with water during N dry–wet cycles; and M*_N_*_0_ is the mass of the specimen when it is dry during N dry–wet cycles.

(1) $$W_{N}=\frac{M_{N1}-M_{N0}}{M_{N0}}\times 100\%$$

Figure 2 illustrates that the water absorption of Pisha sandstone is in the range of 4–5.5%, which is larger than the general rock material. This conclusion indicates that Pisha sandstone is a porous rock with a significant effect of water on it and poor engineering properties. With the increase of the number of wet–dry cycles, the water absorption rate of Pisha sandstone exhibits a gradually decreasing trend. This phenomenon can be attributed to the filling of pores and fissures inside specimens by small-sized clay minerals produced by the water–rock action, resulting in less space to accommodate water molecules in the specimen. Moreover, the clay minerals generated gradually fill the pores and cracks inside the specimen, resulting in poor penetration of water molecules inside specimens.

In addition, the decline rate of water absorption of Pisha sandstone is larger in the early stage of dry–wet cycles (≤2 cycles). Compared with the previous cycle, the water absorption rate of specimens after one cycle and two cycles decreased by 8.52% and 14.29%, respectively. This result indicates that the wet–dry cycling action has a significant effect on the water absorption rate of Pisha sandstone at the early stage of wet–dry cycling. The decline rate of water absorption rate is lower in the late stage of the dry–wet cycles. Compared with the previous cycle, the water absorption rate of the specimens declined by 1.21%, 1.71%, and 1.24% after three cycles, four cycles, and five cycles, respectively.

#### 2.1.3. Stress–Strain Curve

**Figure 3:**
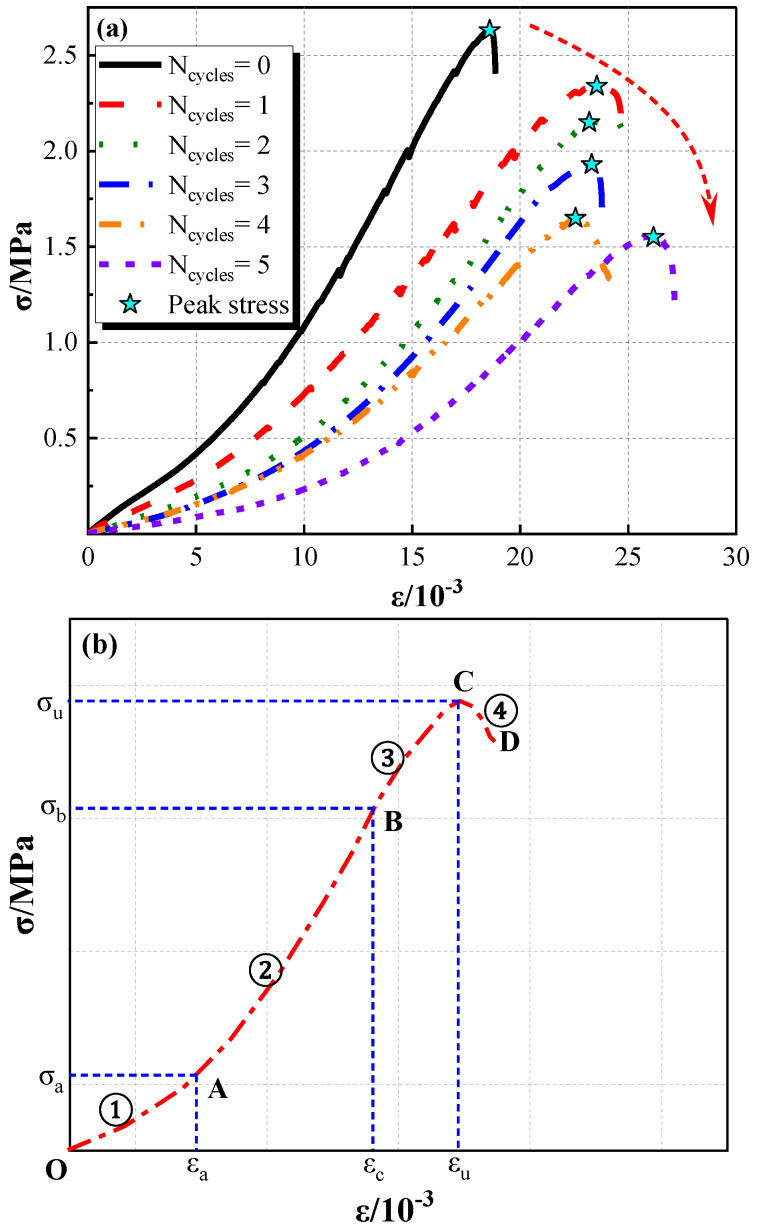
Results of uniaxial compression test: (**a**) stress–strain curve; and (**b**) simplified illustration.

A series of uniaxial compression experiments were carried out to investigate the stress–strain curves of various specimens with different wet–dry cycles. The loading rate is 1 mm/min, and the results are shown in Figure 3.

Figure 3a illustrates that the wet–dry cycle has a significant effect on the uniaxial compressive strength. With the increase of wet–dry cycles, the peak stress (σ_u_) of Pisha sandstone gradually decreases. Compared to the initial specimens, the peak stress of specimens after one cycle, two cycles, three cycles, four cycles, and five cycles dropped by 11.03%, 18.25%, 26.62%, 37.26%, and 41.06%, respectively. The physical and chemical reactions during the wet and dry cycles destroy the original particles and break the bond between the particles, which leads to the reduction of compressive strength. On the contrary, the peak strain (ε_u_) of specimens exhibited a positive correlation with the wet–dry cycles. The peak strain (ε_u_) of specimens after 5 cycles is 1.41 times that of initial specimens. In addition, the trend of the stress–strain curves of all specimens is consistent. 

Figure 3b indicates that the stress–strain curve of specimens can be divided into four stages, including the compaction stage (OA), elastic stage (AB), plastic stage (BC), and descending stage (CD). In the compaction stage (OA), the loose internal structure of Pisha sandstone gradually densely compacts when subjected to low loadings, and the deformation at this stage is plastic deformation. Then, it enters the elastic stage (AB), the stress and strain show a linear relationship. When the stress exceeds the elastic limit stress (σ_b_), the specimen enters the plastic stage (BC), and cracks begin to occur on the surface of the specimen. Finally, when the stress reaches its peak, the stress drops rapidly, and the specimen is completely broken. It should be mentioned that with the increase of dry and wet loading cycles, the strain in the compaction stage (OA) increases significantly. This phenomenon can be attributed to the gradual increase of cracks and pores in the specimen as the number of cycles increases. Meanwhile, a similar phenomenon has emerged in the plasticity phase.

#### 2.1.4. Elastic Modulus

**Figure 4:**
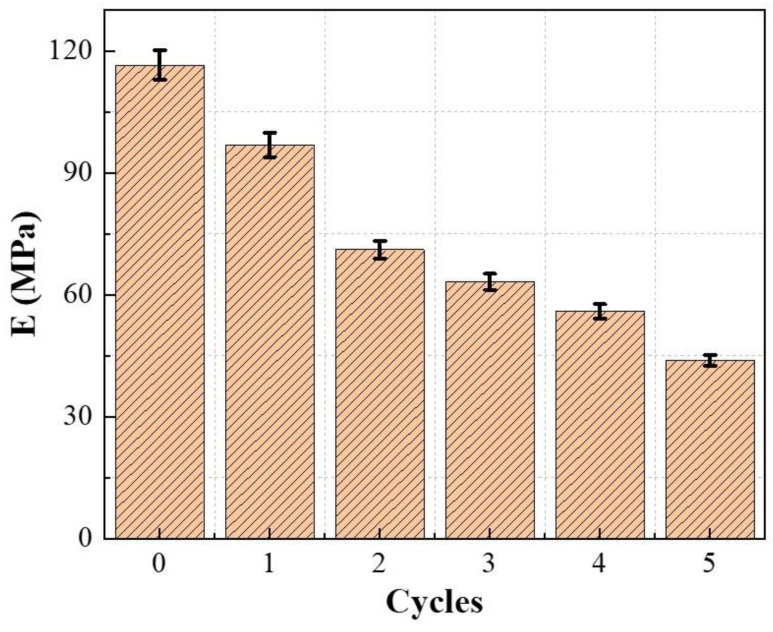
Elastic modulus of specimens at different wet–dry cycles.

Elastic modulus is an essential parameter for the deformation and stability analysis of Pisha sandstone, representing the ability of materials to resist deformation. The secant elastic modulus method is employed to investigate the elastic modulus of specimens after different dry–wet cycles. The elastic modulus of specimens at different wet–dry cycles are listed in Figure 4.

Figure 4 demonstrates that with the increase of wet–dry cycles, the elastic modulus of Pisha sandstone at different wet–dry cycles gradually decreases. The elastic modulus of the initial specimens (0 cycles) is 1.20 times, 1.64 times, 1.84 times, 2.08 times, and 2.66 times that of specimens after one cycle, two cycles, three cycles, four cycles, and five cycles, respectively. As the number of wet–dry cycles increases, the pores and cracks in the specimens gradually increase leading to the reduction of the resistance to deformation.

#### 2.1.5. Fracture Energy

**Figure 5:**
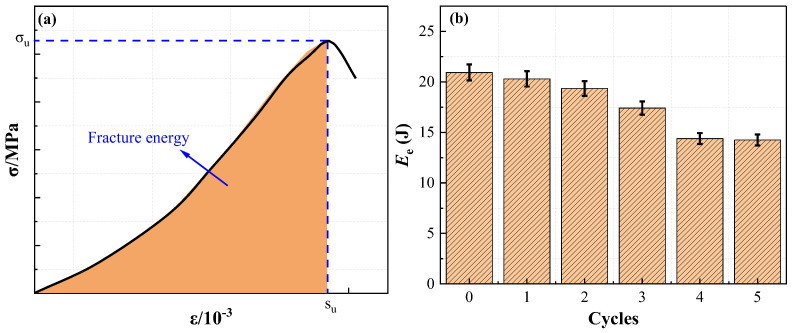
Fracture energy of specimens: (**a**) simplified illustration; and (**b**) fracture energy of specimens at different wet-dry cycles.

The fracture energy (E_e_) was employed to evaluate the energy absorption ability of specimens at different wet–dry cycles. It can be calculated by integrating the area of nominal stress–strain curves as per Equation (2), ranging from 0 to its peak strain (s_u_). The simplified illustration and fracture energy of specimens at different wet–dry cycles are displayed in Figure 5.

(2) $$E_{e}=\int_{0}^{\mathrm{Su}}\sigma \left(s\right)\mathrm{ds}$$

Figure 5 indicates that the fracture energy exhibits a negative correlation with wet–dry cycles. The fracture energy of the initial specimens (0 cycles) is 1.03 times, 1.08 times, 1.20 times, 1.45 times, and 1.47 times that of specimens after one cycle, two cycles, three cycles, four cycles, and five cycles, respectively. This phenomenon can be due to the fact that with the increasing number of wet–dry cycles, the damage inside the specimens gradually increases, which leads to the reduction of the fracture energy. It is noteworthy that the fracture energy of specimens decreases slowly in the early wet–dry cycle.

### 2.2. Mineral Compositions

**Figure 6:**
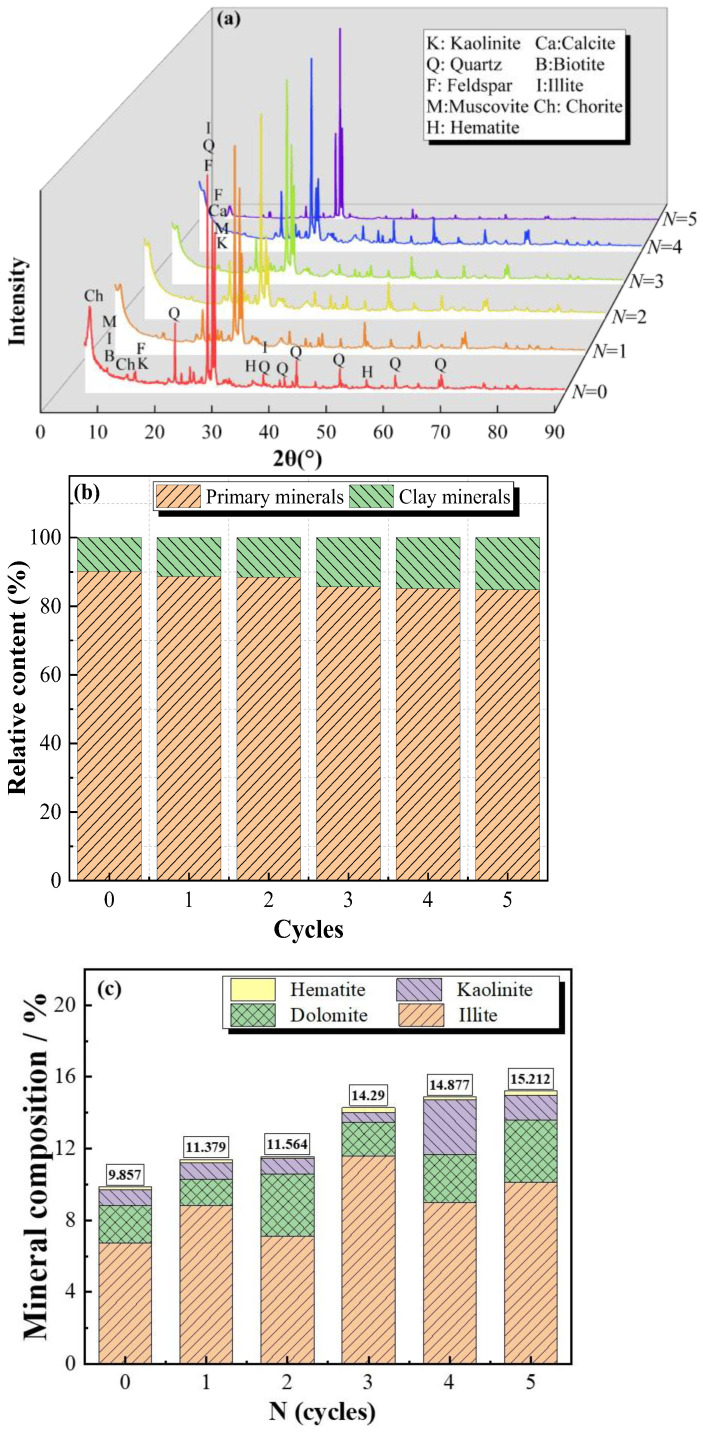
XRD results of specimens at different wet–dry cycles: (**a**) XRD; (**b**) minerals; and (**c**) primary clay minerals.

The XRD technique was applied to determine the mineral types and relative contents of Pisha sandstone specimens after different wet–dry cycles, and the experimental results are depicted in Figure 6. The diffraction peak intensity of the main minerals of Pisha sandstone under different wet–dry cycles is listed in Table 1.

Figure 6 illustrates that Pisha sandstone mainly consists of kaolinite, quartz, feldspar, muscovite, hematite, calcite, biotite, illite, and chlorite, among which the diffraction peaks of quartz (2θ = 26.6°) and feldspar (2θ = 27°) are the most intense. Quartz, feldspar, calcite, and biotite are classified as primary minerals, and kaolinite, muscovite, hematite, chlorite, and illite are regarded as clay minerals. It can be seen from Figure 6b that with the increase of wet–dry cycles, the relative content of primary minerals gradually decreases. Compared to the initial specimens, the relative content of primary minerals after five wet–dry cycles declined by 5.94%. On the contrary, the relative content of clay minerals in samples exhibited the opposite trend. The relative content of clay minerals of the initial samples (0 cycles) is 1.15 times, 1.17 times, 1.45 times, 1.51 times, and 1.54 times that of samples after one cycle, two cycles, three cycles, four cycles, and five cycles, respectively. This is attributed to the decomposition of easily weathering feldspar, calcite, and muscovite into clay minerals during the wet–dry cycles [22]. Furthermore, the relative content of clay minerals increased rapidly in the early stage (≤2) and developed slowly in the later stage of wet–dry cycles.

### 2.3. Microstructure Analysis

**Figure 7:**
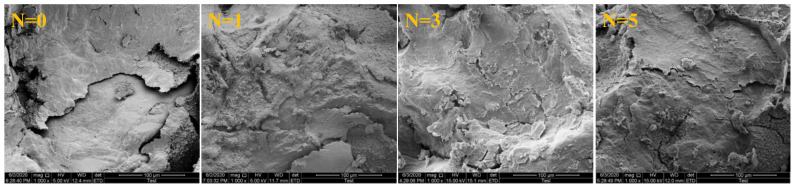
SEM results of specimens at different wet–dry cycles (1000×).

The microstructure changes of specimens at different wet–dry cycles were investigated by SEM, as shown in Figure 7, in which the N represents the number of wet–dry cycles.

Figure 7 revealed that the Pisha sandstone grains of initial samples were smooth and flat, and the direct boundary between grains was clear. With the increase of wet–dry cycles, the surface of Pisha sandstone particles became rough and porous, and the boundary between particles was gradually filled. Under water–rock interaction, the Pisha sandstone was subject to physical and chemical weathering, resulting in reduced smoothness and density. Moreover, the size of Pisha sandstone particles also gradually became smaller. This is because during the wet–dry cycle, primary minerals with larger particle sizes gradually decomposed into secondary minerals with smaller particle sizes [18,23].

### 2.4. Porosity Analysis

#### 2.4.1. Porosity and Distribution

**Figure 8:**
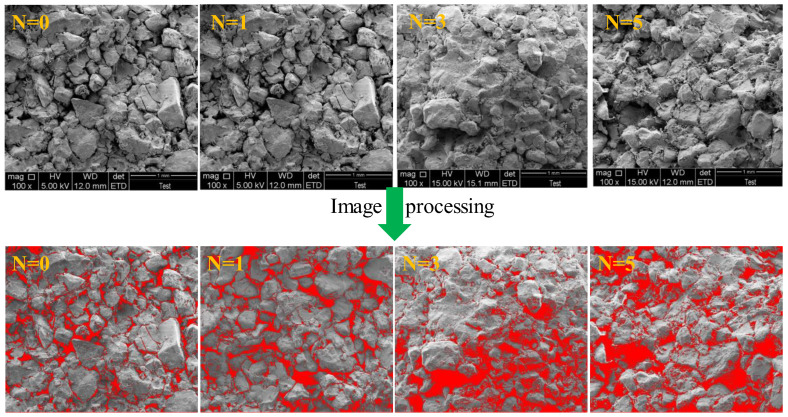
Image processing of specimens at different wet-dry cycles.

**Figure 9:**
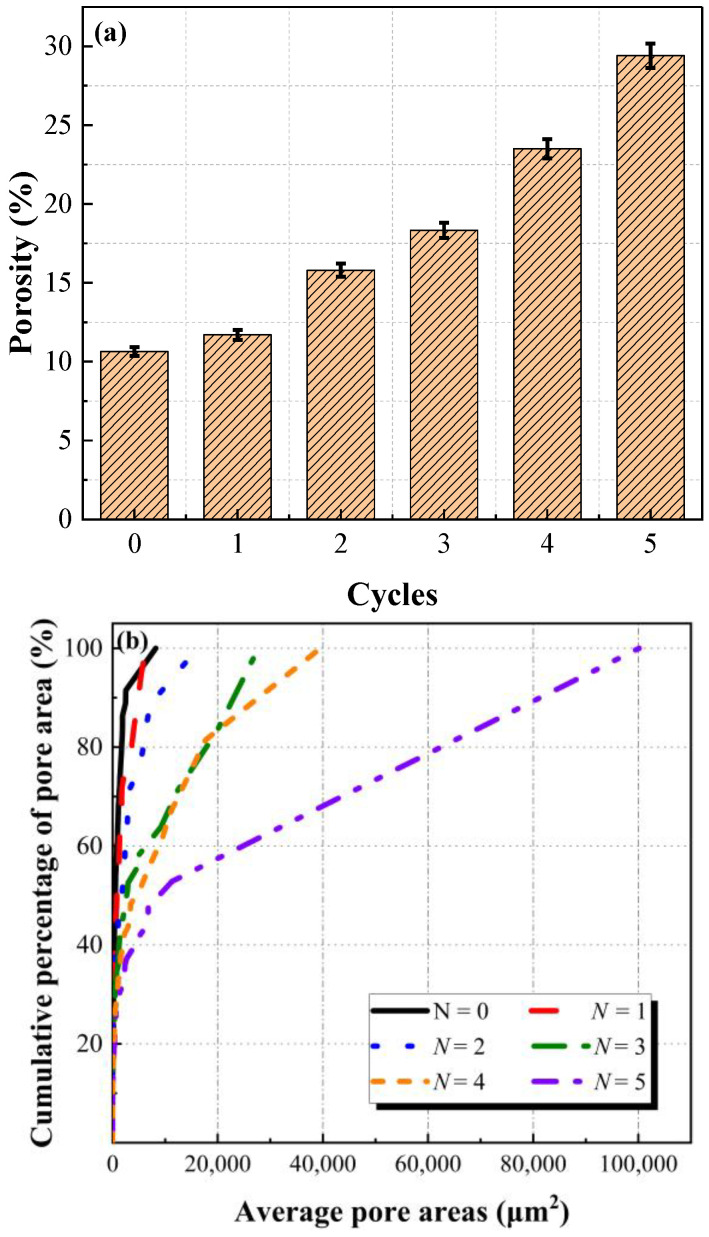

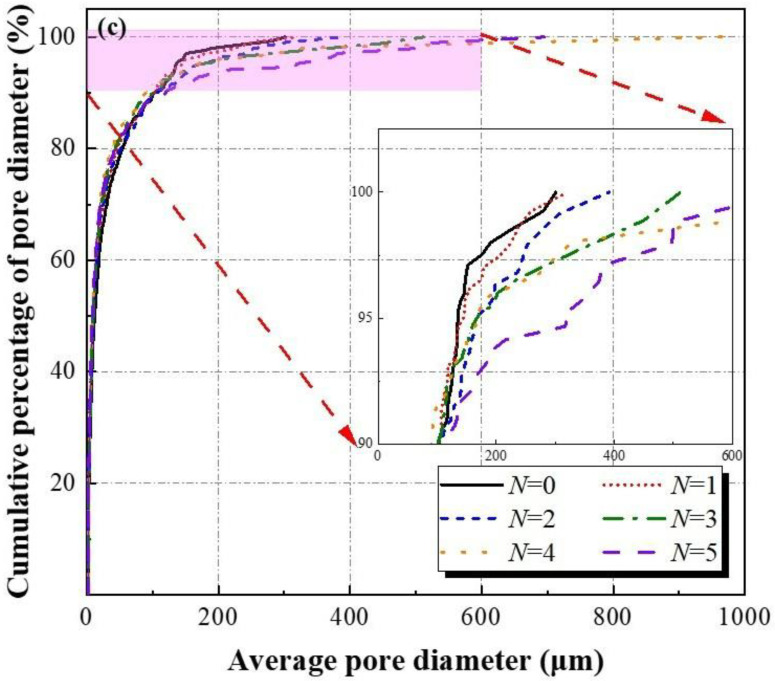
Porosity of specimens at different wet–dry cycles: (**a**) porosity; (**b**) average pore area; and (**c**) average pore diameter.

A pixel-based image processing algorithm base on the SEM results was employed to compare and analyze the porosity and distribution of specimens at different wet–dry cycles [16,24]. In order to provide more analysis objects and meet the statistical requirements, the SEM results with a magnification of 100 times were selected. In addition, each group of six SEM images was chosen to reduce errors. The image processing of specimens at different wet–dry cycles was given in Figure 8, wherein the gray and red contrast in the images showed the density distribution of the voids. With the increase of wet–dry cycles, the red area gradually increased, indicating that the defect area and porosity of samples increased. It should be mentioned that the defect area started to connect and became huge pores when the number of wet–dry cycles exceeded three. The porosity and distribution of samples after different wet–dry cycles are illustrated in Figure 9.

Figure 8 and Figure 9 demonstrate that the porosity of samples exhibits a positive correlation with wet–dry cycles. Compared to the initial specimens, the porosity of specimens after one cycle, two cycles, three cycles, four cycles, and five cycles increased by 9.96%, 48.50%, 72.27%, 120.86%, and 176.32%, respectively. It is noteworthy that the porosity of specimens grows slowly before two cycles and then develops rapidly afterward. Furthermore, with the increase of wet–dry cycles, the average pore area and average pore diameter of samples gradually increase. The cumulative percentage of pore area in each group of specimens with an average pore area less than 10,000 μm^2^ is 67.67%, 56.31%, 46.34%, 38.03%, 29.11%, and 26.46%, respectively. The cumulative percentage of pore diameter in each group of specimens with an average pore diameter less than 100 μm is 91.70%, 90.09%, 89.89%, 89.86%, 89.31%, and 88.95%, respectively. Thereby, the average pore area is more sensitive to wet–dry cycles than the average pore diameter. After five cycles, the cumulative proportion of average pore area decreases by 43.21%, while the cumulative proportion of average pore diameter reduces by 2.75%. This is because under the water–rock interaction, small pores gradually connect to form large pores, and new small pores are generated.

#### 2.4.2. Correlation between Porosity and Strength

A prediction model is proposed to represent the correlation between uniaxial compressive strength and porosity, as shown in Equation (3). Among them, σ_c_ and P are the uniaxial compressive strength (MPa) and porosity (%) of specimens at different wet–dry cycles, respectively.

(3) $$\sigma_{c}=\frac{8.31}{\sqrt{p}}$$

**Figure 10:**
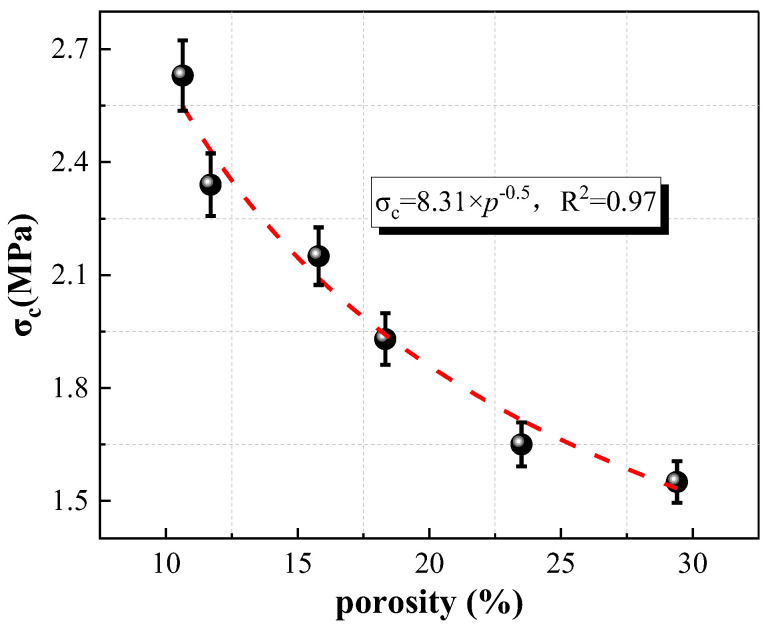
Relationship between uniaxial compressive strength and porosity.

Figure 10 displays the correlation between uniaxial compressive strength and porosity, and Table 2 shows the error analysis of experimental value (σ_E_) and prediction (σ_Pre_) value. The uniaxial compressive strength and porosity exhibit a negative correlation trend. When the porosity of samples increases from 10.64% to 29.40%, the uniaxial compressive strength of specimens decreases by 41.06%. This is because the pores and cracks inside specimens gradually increase along with the increases of wet–dry cycles, leading to a reduction in macro mechanical strength. In addition, the prediction model proposed (Equation (3)) in this work is in good agreement with the experimental results. The errors of all specimens at different wet–dry cycles were within the range of −3.89–3.13% indicating the feasibility and accuracy of Equation (3) as a prediction model for the uniaxial compressive strength–porosity.

#### 2.4.3. Correlation between Porosity and Clay Mineral Content

**Figure 11:**
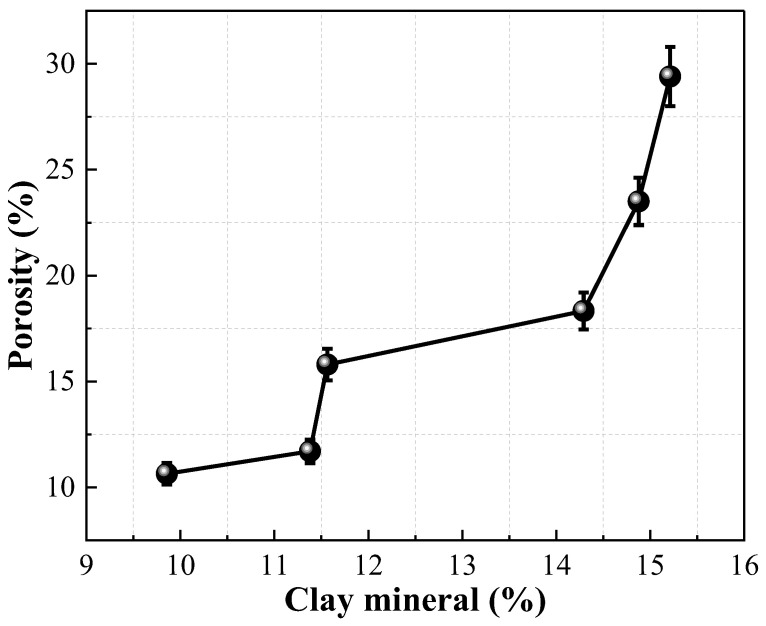
Relationship between porosity and clay mineral content.

**Figure 12:**
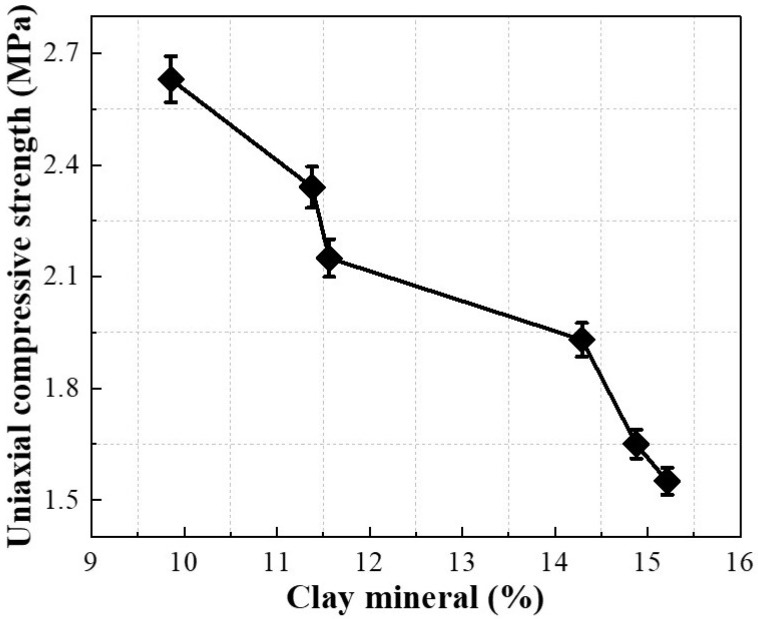
Relationship between clay mineral content and uniaxial compressive strength.

The correlation between porosity and clay mineral content of Pisha sandstone at different wet–dry cycles is depicted in Figure 11. With the increase of clay mineral content, the porosity of specimens gradually increased. When the clay mineral content increased from 9.86% to 15.21%, the porosity of samples grew from 10.64% to 29.40%, rising by 176.32%. This phenomenon is attributed to the decomposition of primary minerals into clay minerals during the wet–dry cycles, which increases the cracks and pores inside the specimens. Figure 12 illustrates the correlation between clay mineral content and the uniaxial compressive strength of Pisha sandstone at different wet–dry cycles. The uniaxial compressive strength of specimens exhibited a negative correlation with the clay mineral content. When the clay mineral content increased from 9.86% to 15.21%, the uniaxial compressive strength of samples decreased from 2.63 MPa to 1.55 MPa, a decrease of 41.06%. This is because the decomposition of primary minerals into clay minerals increases the internal defects of specimens, leading to a decrease in macroscopic mechanical properties.

#### 2.4.4. Discussion

During the dry–wet cycles, significant changes were found in the occurrence state and water content in the Pisha sandstone. In the wetting stage, the water content in the Pisha sandstone gradually increases, which promotes the dissolution of feldspar, calcite, and muscovite [25,26]. The cohesive force inside the Pisha sandstone gradually decreases, and the porosity gradually increases. Moreover, the easily weathered feldspar, calcite, and muscovite gradually decomposed into clay minerals with smaller particle sizes, which further increased the porosity inside specimens. In the drying stage, the water content inside the Pisha sandstone gradually decreases, and the internal components begin to shrink due to water loss, further reducing the cohesive force and increasing the porosity. Sumner investigated the effect of water content on the physical properties of sandstone under the action of wet–dry cycles [27]. Li studied the effect of wet–dry cycles on mechanical parameters, such as deformation, strength, and damage morphology, of sandstone [19]. Wu examined the deterioration law of mechanical properties of remolded soft sandstone under wet–dry cycles. Although many scholars have carried out studies on the physical properties, chemical properties, and mechanical properties of Pisha sandstone in different environments, there are few reports on the mineral composition, microstructure, and void fraction of Pisha arsenic sandstone under wet–dry cycles. Therefore, the change law of mineral composition, microstructure, porosity, and mechanical strength of Pisha sandstone was analyzed, which provides a reference for the analysis of the stability of new Pisha sandstone slopes, the prediction of Pisha sandstone life, and treatment measures.

## 3. Experimental Design

### 3.1. Sample Selection

The Pisha sandstone samples were collected from the Pisha sandstone area (39.46° N, 110.49° E) in the Nuanshui Country, Junger Banner, Ordos City, Inner Mongolia, which was one of the typical soil erosion regions [28]. Due to heavy weathering and the loose layer structure of Pisha sandstone, the surficial layer of Pisha sandstone is not suitable for sampling. Therefore, the virgin Pisha sandstone was derived underground with a depth of 2.5 m to ensure stability, homogeneity, and low weathering degree [22]. Pisha sandstone blocks with small surface joint development, no delamination, and cracks were selected. To facilitate the preparation of specimens, the size of the Pisha sandstone blocks should not be less than 400 mm × 400 mm × 400 mm. A large number of bubble columns were applied to wrap the Pisha sandstone blocks to reduce disturbance and damage during transport.

In addition, further screening of the primary samples was carried out to reduce the dispersion of the experimental results and ensure the validity and comparability of the experiment. Therefore, the mineral composition screening method and microscopic morphology screening method are adopted in the current test. The mineral composition of all samples was determined by XRD. Considering that feldspar, calcite, and muscovite decompose into clay minerals during the wet–dry cycle tests, the content of primary minerals and clay minerals was employed as an indicator for screening samples. When the content of any mineral in a sample differs from the average value by more than 5%, the sample will be excluded. The main indicators include the content of feldspar, calcite, and muscovite; the total amount of primary minerals; and the total amount of clay minerals. Moreover, a super depth-of-field three-dimensional micro-observation instrument was adopted to observe the microscopic morphology of all samples. When there is a significant difference in the microscopic morphology (pores and cracks) of the sample, the sample will be excluded. 

**Figure 13:**
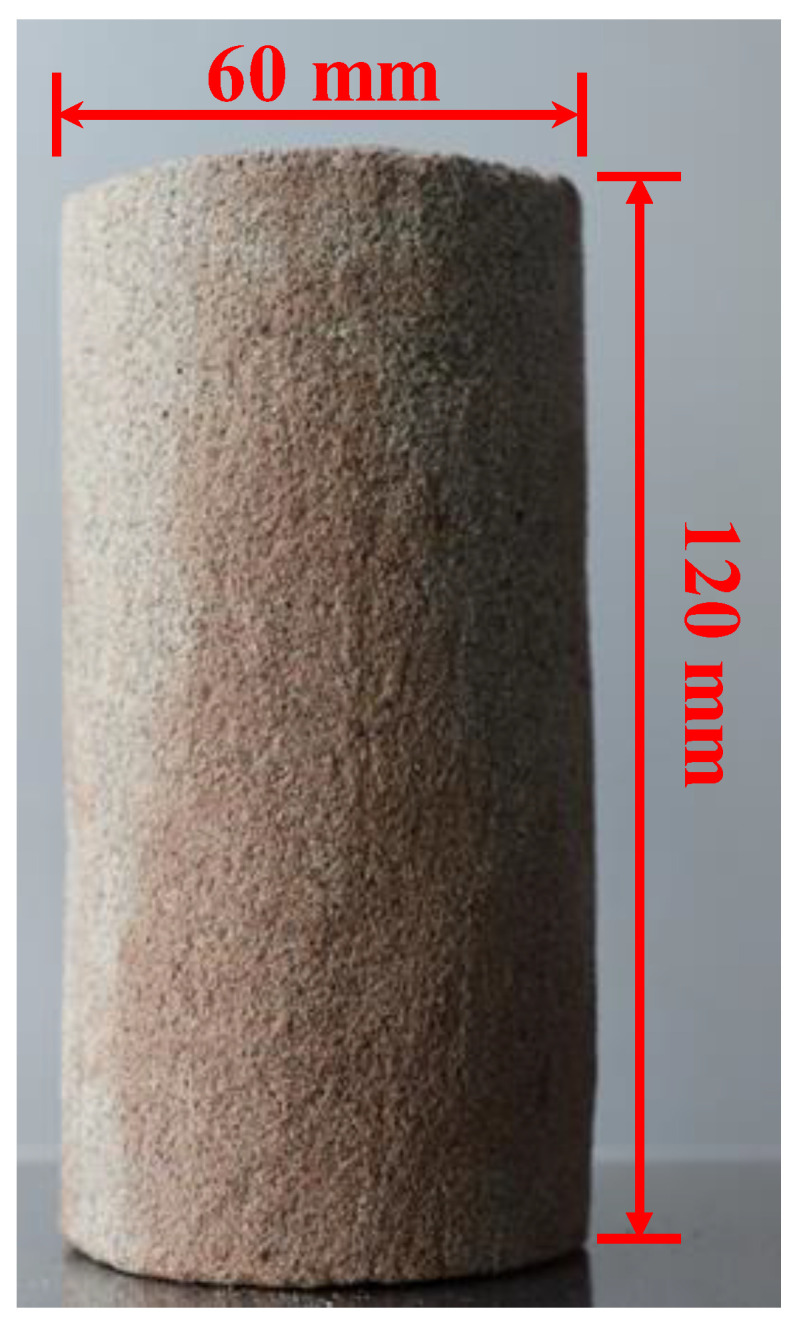
Dimensions of Pisha sandstone specimens.

According to the Standard for Geotechnical Test Methods (GB/T 50123-2019) [29], 36 cylinders with a diameter of 60 mm and a height of 120 mm (see Figure 13) were prepared and divided into six groups on average. The average values of the physical properties of the initial Pisha sandstone specimens are listed in Table 3.

### 3.2. Wet-Dry Cycling Test

**Figure 14:**
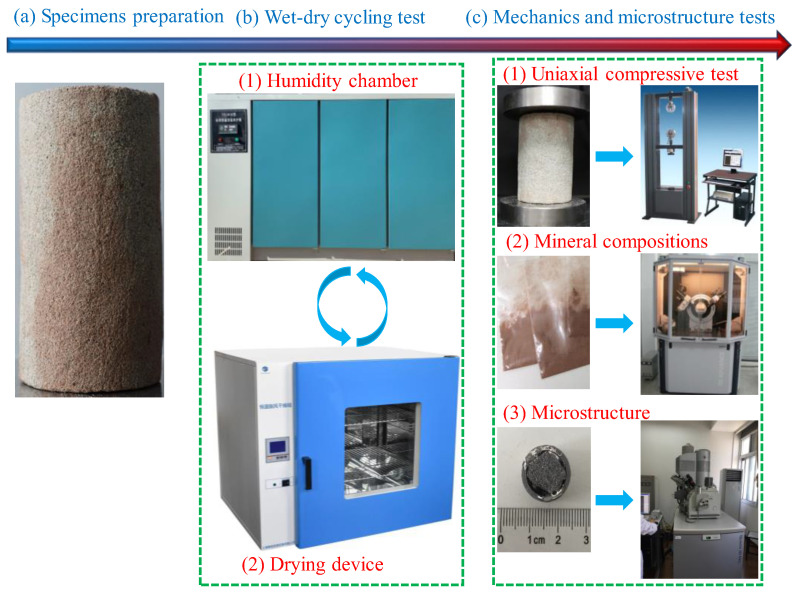
Procedure of the wet–dry cycle test.

Figure 14 displays the procedure of the wet–dry cycling test. The wet–dry cycling test method was improved by considering “water absorption in high-humidity environment–drying with device” as one cycle, which enhances the operability of wet–dry cycle experiments and also conforms to the actual situation in the field [30,31]. The experiment process can be briefly summarized as follows: firstly, specimens were placed into a constant temperature (20 ± 2 °C) and humidity (95% ± 1%) curing tank to absorb moisture until reaching stable weights, which took about 24 days. Secondly, specimens were dried in a drying oven for 24 h at 105 °C. Lastly, samples were kept in a drying container and prepared for further experiments or wet–dry cycles. In summary, each cycle is roughly about 25 days. Further experiments mainly include the uniaxial compression experiment, X-ray diffraction analysis, and SEM observation. The specimens and equipment are shown in Figure 14. In addition, the appearance, mass (saturated with water and dry), mineral composition, and microstructure of specimens after each wet–dry cycle need to be recorded and analyzed.

### 3.3. Test Equipment

A YDW-10 universal testing machine was employed to investigate the uniaxial compression experiment of Pisha sandstone. The XRD result was obtained by the D8-Discover X-ray diffractometer from Bruker, Germany. Diffraction patterns were obtained at 2θ from 5° to 90° with a scanning rate of 0.15 s/step, and the step width was 0.02°. A VHX-2000E super depth-of-field three-dimensional micro-observation instrument was used to observe the surface morphology of the selected samples. A Scanning Electron Microscope (SEM) was adopted to investigate the microstructure of Pisha sandstone specimens under different wet–dry cycles. The Quanta 3D FEG field emission environment scanning electron microscope of Germany FEI company was adopted, with an acceleration voltage of 200 V–30 kV, an electron beam current of 200 nA, magnification of 30–1,280,000, and environment scanning modulus of 10–4000 Pa.

## 4. Conclusions

(1) With the increase of wet–dry cycles, the mechanical properties of Pisha sandstone gradually decrease. After five wet–dry cycles, the uniaxial compressive strength, elastic modulus, and fracture energy of specimens were reduced by 41.06%, 62.39%, and 31.92%, respectively. The stress–strain curves of specimens can be divided into four stages: the compaction stage, elastic stage, plastic stage, and descending stage. In addition, the failure mode of the specimen changes from inclined shear failure to peel failure.
(2) With the increase of wet–dry cycles, the relative content of primary minerals gradually decreased, while the relative content of clay minerals gradually increased. Compared to the initial specimens, the relative content of primary minerals after five wet–dry cycles declined by 5.94%, and the relative content of clay minerals after five wet–dry cycles increased by 54.33%. Furthermore, the relative content of clay minerals increased rapidly in the early stage (≤2) and developed slowly in the later stages of wet–dry cycles.
(3) Under water–rock interaction, the Pisha sandstone was subject to physical and chemical weathering, and the surface of the Pisha sandstone particles became rough and porous. Additionally, the porosity of samples exhibits a positive correlation with wet–dry cycles. Compared to the initial specimens, the porosity of specimens after five wet–dry cycles increased by 176.32%.
(4) The uniaxial compressive strength and porosity exhibit a negative correlation trend. When the porosity of samples increases from 10.64% to 29.40%, the uniaxial compressive strength of specimens decreases by 41.06%. Moreover, the prediction model proposed in this work is in good agreement with the experimental results.

In addition, the differences in mineral composition, mechanical strength, microstructure, and voids between the initial specimens and the specimens after different wet and dry cycles were systematically analyzed. The results provide a reference for the analysis of the stability of new Pisha sandstone slopes, the prediction of Pisha sandstone life, and treatment measures.

## Figures and Tables

**Table 1:** Diffraction peak intensity of main minerals of Pisha sandstone (%).

Minerals	d (A)	N = 0	N = 1	N = 2	N = 3	N = 4	N = 5
Ch	14.98	37.93	33.59	37.45	28.61	31.87	6.37
M, B, I	9.81	11.82	11.84	13.81	10.76	12.69	1.53
Ch	7.01	8.92	8.814	10.08	7.86	9.19	1.64
F, K	6.31	10.25	8.11	9.17	7.19	8.43	1.35
Q	4.22	32.23	11.50	12.11	9.55	10.56	1.69
Q, F, I	3.33	100.00	100.00	100.00	100.00	100.00	100.00
F, Ca	3.22	79.84	38.70	19.91	24.54	17.85	51.56
M, K	3.17	73.68	17.48	11.62	14.70	14.06	25.43
H	2.57	5.21	8.22	8.72	6.83	8.66	1.27
Q, I	2.45	8.94	9.65	9.62	7.60	9.89	1.37
Q	2.23	7.53	5.69	6.26	5.20	6.59	0.92
Q	2.12	15.25	8.92	8.16	6.95	10.98	2.14
H	1.67	6.31	7.49	6.55	6.18	6.68	1.37
Q	1.54	8.40	10.43	9.17	7.89	9.98	2.41
Q	1.37	7.11	8.45	8.17	10.01	10.53	1.042

K—Kaolinite; Q—Quartz; F—Feldspar; M—Muscovite; H—Hematite; Ca—Calcite; B—Biotite; I—Illite; Ch—Chorite; d—the spacing of crystal planes; N—the number of wet–dry cycles.

**Table 2:** Error analysis.

N	σ_E_ (MPa)	σ_Pre_ (MPa)	σ_E_/σ_Pre_
0	2.63	2.55	1.03
1	2.34	2.43	0.96
2	2.15	2.09	1.03
3	1.93	1.94	0.99
4	1.65	1.71	0.96
5	1.55	1.53	1.01

Where N is the number of wet-dry cycles, σ_E_ is the experimental value of uniaxial compressive strength, and σ_Pre_ is the prediction value of uniaxial compressive strength.

**Table 3:** The average values of the physical properties of the initial Pisha sandstone specimens.

Diameter (mm)	Height (mm)	Mass (g)	Water Content (%)	Density (g/cm^3^)
60.00	120.00	766.13	5.23	2.26

## Data Availability

The data presented in this study are available in the article. This manuscript has not been published or submitted in whole or in part to any other journal. The datasets used and/or analyzed during the current study are available from the corresponding author on reasonable request.

## Sample Availability

Samples of the compounds are available from the authors.

## References

[1] Ma W., Zhang X. (2016). Effect of Pisha sandstone on water infiltration of different soils on the Chinese Loess Plateau. J. Arid. Land.

[2] Li C., Zhang T., Wang L. (2014). Mechanical properties and microstructure of alkali-activated Pisha sandstone geopolymer composites. Constr. Build. Mater..

[3] Wang Y., Wu Y., Kou Q., Chang Y., Zhang R. (2007). Definition of distribution range and type division of soft sandstone. Sci. Soil Water Conserv..

[4] Zhao C., Gao J., Huang Y., Wang G., Xu Z. (2017). The contribution of Astragalus adsurgens roots and canopy to water erosion control in the water–wind crisscrossed erosion region of the Loess Plateau, China. Land Degrad. Dev..

[5] Zhang P., Yao W., Xiao P., Liu G., Yang C. (2022). Study on the interaction and superposition effect of multi-dynamic erosion in the soft sandstone area of the Yellow River basin. J. Hydraul. Eng..

[6] Ziadat F.M., Taimeh A.Y. (2013). Effect of rainfall intensity, slope, land use and antecedent soil moisture on soil erosion in an arid environment. Land Degrad. Dev..

[7] Liang Z., Liu H., Zhao Y., Wang Q., Gao H. (2019). Effects of rainfall intensity, slope angle, and vegetation coverage on the erosion characteristics of Pisha sandstone slopes under simulated rainfall conditions. Environ. Sci. Pollut. Res..

[8] Yang C., Liu Q., Qu F., Zhao Y., Wu Z. (2019). Experimental study on weathering characteristics of Pisha sandstone. Adv. Sci. Technol. Water Resour..

[9] Xiao P., Yao W., Liu H. (2014). Research progress and harnessing method of soil and water loss in Pisha sandstone region. Yellow River.

[10] Wang Y., Wu Y., Min D., Chang Y., Zhang R. (2007). Investigation on measures for soil erosion in soft rock area. Glob. Seabuckthorn Res. Dev..

[11] Li J. (2016). Study on Influence Factors of Slope Erosion with Scouring Experiment in Pisha Sandstone Region. Master’s Thesis.

[12] Wang Q. (2016). Erosion Study on Pisha Sandstone Slope under Simulated Rainfall Conditions. Master’s Thesis.

[13] Wang Q., Sun X., Liu Y., Zhang G., Shi J., Ye H. (2013). Indoor modeling the effect of water-rock interaction on the weathering and erosion of Pi-sandstone. Yellow River.

[14] Su T., Zhang X., Zhao H. (2011). Study on hydraulic characteristics of slope runoff in Pisha sandstone region. J. Northwest A F Univ. (Nat. Sci. Ed.).

[15] Su T., Zhang X. (2012). Hydraulic Characteristics of Steep Slope Runoff of Pisha Sandstone. J. Soil Water Conserv..

[16] Shi J., Ye H., Wang Q., Sun Y. (2009). Effect of water-rock interaction on the weathering and erosion of Pi-sandstone, Southern Inner Mongolia, China. Geoscience.

[17] Nara Y., Morimoto K., Yoneda T., Kaneko K. (2009). Effect of the Humidity on Subcritical Crack Growth in Sandstone. J. Mmij.

[18] Yang X., Wang J., Zhu C., He M., Gao Y. (2019). Effect of wetting and drying cycles on microstructure of rock based on SEM. Environ. Earth Sci..

[19] Wu S., Li X., Chang P., Li M., Chne S. (2018). Experimental study on mechanical properties of red Pisha-sandstone under dry–wet cycles in Ordos. J. Drain. Irrig. Mach. Eng..

[20] Wu S., Li X., Chang P., Zhang Y., Guo L. (2019). Experimental study on mechanical properties of red Pisha-sandstone cement soil under wetting-drying cycles. J. Drain. Irrig. Mach. Eng..

[21] Ma W., Ding Z., Wu Z., Liang Z., Yang C. (2018). Mechanical properties and meso-mechanism of Pisha sandstone with W-OH solidified under drying-wetting cycles. Bull. Soil Water Conserv..

[22] (2019). Standard for Geotechnical Test Method.

[23] Ma W., Gao W., Guo S., Zhao Y., Wu Z., Yang C. (2020). Evaluation and improvement on the freeze-thaw durability performance of the polyurethane stabilized Pisha sandstone for water and soil conservation. Cold Reg. Sci. Technol..

[24] Zhao Y., Yang C., Li K., Qu F., Yan C., Wu Z. (2022). Toward understanding the activation and hydration mechanisms of composite activated coal gangue geopolymer. Constr. Build. Mater..

[25] Fu Y. (2010). Study on Water-Rock Interaction with Cyclic Drying-Wetting Effect on Rock. Master’s Thesis.

[26] Summer P., Loubser M. (2008). Experimental sandstone weathering using different wetting and drying moisture amplitudes. Earth Surf. Process. Landf..

[27] Li K., Zheng D., Huang W. (2013). Neural network simulation of mechanical properties and constitutive model of sandstone under dry–wet cycles. Rock Soil Mech..

[28] Liang Z., Wu Z., Yao W., Noori M., Yang C., Xiao P., Leng Y., Deng L. (2019). Pisha sandstone: Causes, processes and erosion options for its control and prospects. Int. Soil Water Conserv. Res..

[29] Liu X., Liu Q., Huang S., Liu B., Liu J. (2021). Effects of cyclic wetting-drying on the mechanical behavior and improved damage model for sandstone. Mar. Georesour. Geotechnol..

[30] Huang S., He Y., Liu X., Xin Z. (2021). Experimental investigation of the influence of dry-wet, freeze-thaw and water immersion treatments on the mechanical strength of the clay-bearing green sandstone. Int. J. Rock Mech. Min. Sci..

[31] Ye H., Shi J., Li X., Hou H., Shi Y., Chen Y. (2006). The effect of soft rock lithology upon its anti erosion ability. Acta Geosci. Sin..

